# The Goto-Kakizaki rat is a spontaneous prototypical rodent model of polycystic ovary syndrome

**DOI:** 10.1038/s41467-021-21308-y

**Published:** 2021-02-16

**Authors:** Camille Bourgneuf, Danielle Bailbé, Antonin Lamazière, Charlotte Dupont, Marthe Moldes, Dominique Farabos, Natacha Roblot, Camille Gauthier, Emmanuelle Mathieu d’Argent, Joelle Cohen-Tannoudji, Danielle Monniaux, Bruno Fève, Jamileh Movassat, Nathalie di Clemente, Chrystèle Racine

**Affiliations:** 1 Sorbonne Université-INSERM, Centre de Recherche Saint-Antoine (CRSA), Paris, France; 2 Institut Hospitalo-Universitaire ICAN, Paris, France; 3 Université de Paris, BFA, UMR 8251, CNRS, F-75013, Paris, France; 4 Assistance Publique des Hôpitaux de Paris, Hôpital Saint-Antoine, Département PM2, Paris, France; 5 Assistance Publique des Hôpitaux de Paris, Hôpital Tenon, Service de biologie de la reproduction-CECOS, Paris, France; 6 UMR PRC, INRA, CNRS, Université de Tours, IFCE, Nouzilly, France; 7 Assistance Publique des Hôpitaux de Paris, Hôpital Saint-Antoine, Service Endocrinologie, CRMR PRISIS, Paris, France; 8 Université de Paris, Paris, France

**Keywords:** Physiology, Endocrinology

## Abstract

Polycystic ovary syndrome (PCOS) is characterized by an oligo-anovulation, hyperandrogenism and polycystic ovarian morphology combined with major metabolic disturbances. However, despite the high prevalence and the human and economic consequences of this syndrome, its etiology remains unknown. In this study, we show that female Goto-Kakizaki (GK) rats, a type 2 diabetes mellitus model, encapsulate naturally all the reproductive and metabolic hallmarks of lean women with PCOS at puberty and in adulthood. The analysis of their gestation and of their fetuses demonstrates that this PCOS-like phenotype is developmentally programmed. GK rats also develop features of ovarian hyperstimulation syndrome. Lastly, a comparison between GK rats and a cohort of women with PCOS reveals a similar reproductive signature. Thus, this spontaneous rodent model of PCOS represents an original tool for the identification of the mechanisms involved in its pathogenesis and for the development of novel strategies for its treatment.

## Abstract (web summary)

Although polycystic ovary syndrome is the most common cause of female infertility, its etiology remains poorly understood. Here, the authors report a rat model that spontaneously exhibits the clinical heterogeneity of this syndrome and demonstrate that the phenotype is developmentally programmed.

## Introduction

Polycystic ovary syndrome (PCOS) is the most common cause of female infertility, affecting about 5–10% of women of reproductive age worldwide. The 2003 Rotterdam criteria for the diagnosis of PCOS are based on at least two of the following three features: (1) an irregular ovulatory function (oligomenorrhea or amenorrhea), (2) the evidence of either biological or clinical hyperandrogenism, and (3) the presence of a polycystic ovarian morphology^1^. Most patients with PCOS also exhibit high serum anti-Müllerian hormone (AMH) and luteinizing hormone (LH) levels^2^. In addition, this multifactorial disorder is generally associated with major metabolic disturbances, such as insulin resistance (IR), abdominal adiposity, high cholesterol levels, and high blood pressure with an increased risk of cardiovascular diseases and of type 2 diabetes mellitus (T2DM). Between 28% and 88% of patients with PCOS are obese according to their ethnic background^3^. However, 50% of women with PCOS fulfill the metabolic syndrome criteria regardless of their body mass index (BMI), suggesting that obesity mainly exacerbates their metabolic disturbances.

Despite the high prevalence of PCOS, the etiology of this syndrome is still misunderstood. There is evidence of heritability, as illustrated by the familial clustering of phenotypic features, observed in 20–40% of patients^4^. Several PCOS susceptibility genes or loci have been identified but these only account for a small percentage of women with PCOS and cannot explain the heterogeneity of PCOS phenotypes, suggesting the involvement of other mechanisms^5^. Cumulative evidence now indicates that the reproductive and metabolic disorders observed in women with PCOS might result from developmental alterations during fetal and pre-pubertal life, in accordance with the DoHAD concept (Developmental origins of Health and Desease)^6^. In particular, it is widely accepted, on the basis of clinical studies and of the analysis of various animal models exposed to androgens during prenatal and neonatal life, that hyperandrogenism plays a crucial role in the development of PCOS^4^. A recent hypothesis has suggested that the high AMH levels in pregnant women with PCOS could contribute to the androgenization of their fetuses leading to PCOS in adulthood^7^. Moreover, consistent with the heritability of the PCOS phenotype, Risal et al. ^8^, have recently reported a transgenerational effect of a fetal dihydrotestosterone (DHT) treatment in a PCOS-like mice model. Furthermore, metabolic factors are also likely to contribute to the pathophysiology of PCOS. In particular, supporting a role of IR and associated hyperinsulinemia, various clinical and animal model studies demonstrated that insulin-sensitizing drugs reduce androgen levels and ameliorate both metabolic and reproductive disturbances in women with PCOS^9^.

However, these approaches have not made it possible to determine the timing of PCOS phenotype development, the precise part played by each actor and the underlying mechanisms. Rather, they highlight the great complexity of generating an animal model that mimics the clinical heterogeneity of PCOS and the necessity of having to our disposal spontaneous PCOS animal models. Indeed, only one model, that of a spontaneous hyperandrogenic rhesus monkey, has been shown to exhibit a PCOS-like phenotype but extensive studies are limited in this species^10^.

The inbred Goto-Kakizaki (GK) rat strain is one of the best spontaneous non-obese T2DM animal models, in which obesity can be induced by a high fat diet^11^. Unlike most other genetic models of T2DM, diabetes in GK rats is polygenic. This strain was established by the selection of bred Wistar rats exhibiting glucose intolerance. As early as at fetal age of 16.5 days, GK rats present a fetal loss of pancreatic β cell mass. Later on, GK rats spontaneously exhibit IR, late diabetes complications such as cardiovascular disease and an accumulation of visceral adipose tissue (VAT) associated with the presence of a chronic state of inflammation, hepatic steatosis, and anxiety^12^. It should be noted that all of these disorders are found in women with PCOS. One study alone has reported any modification in the length of the estrus cycle (a longer proestrus and a shorter estrus period) in 6-month-old GK rats^13^.

In this work, we show that female GK rats encapsulates naturally all the reproductive and metabolic hallmarks of lean women with PCOS at puberty and in adulthood. The analysis of their gestation and of their fetuses demonstrates that this PCOS-like phenotype is developmentally programmed. GK rats also develop features of ovarian hyperstimulation syndrome (OHSS) following gonadotropin stimulation. Last, a comparison between GK rats and a cohort of women with PCOS using a multivariate model, reveals a similar reproductive signature. Thus, this spontaneous rodent model of PCOS represents an original tool for the identification of the mechanisms involved in its pathogenesis and for the development of novel strategies for its treatment.

## Results and discussion

Female GK rats used in this study were issued from the Paris colony (GK/Par line). Their characteristics were stable throughout the generations and were similar to those previously published^12^. Nondiabetic Wistar rats from our local colony were used as controls.

### Impaired insulin secretion and glucose tolerance in GK rats

**Fig. 1:**
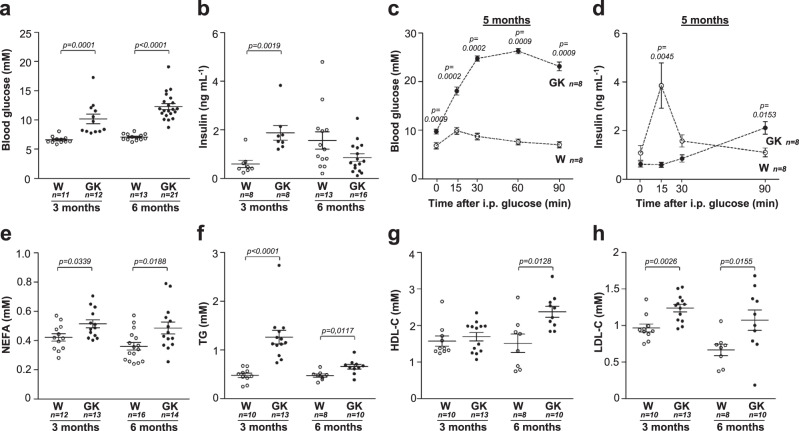
Glucido-lipidic phenotype of Wistar and GK rats. **a**, **b** Assessment of blood glucose (**a**) and plasma insulin levels (**b**) in 3-month-old and 6-month-old Wistar (W, open symbols) and GK (closed symbols) rats. **c**, **d** Measurements of blood glucose (**c**) and plasma insulin levels (**d**) during an intraperitoneal glucose (i.p.) tolerance test performed on 5-month-old Wistar and GK rats. The data are represented as mean ± SEM. **e**–**h** Serum levels of non-esterified fatty acids (NEFA) (**e**), and plasma concentrations of triacylgycerols (TG) (**f**), HDL-cholesterol(-C) (**g**) and LDL-cholesterol (-C) (**h**) at 3 and 6 months. *p*-values are from two-sided Mann–Whitney *U* test for unpaired comparisons (GK vs. Wistar rats). Unless indicated data are represented as scatter blot with mean ± SEM. *n* represents the number of biologically independent animals in each group. Source data are provided as a Source Data file.

In agreement with previous reports^12^, 3-month-old and 6-month-old GK rats exhibited an increase in basal blood glucose levels compared to age-matched Wistar rats (Fig. 1a). Their corresponding insulin levels were increased at 3 months, and, at 6 months, were brought to the insulin levels of Wistar rats (Fig. 1b). These findings suggest that whereas the animals exhibit an IR profile at 3 months, they subsequently shift to an insulinopenic phenotype at 6 months (Fig. 1b), which becomes significant at 18 months^12^. Consistent with a period of IR in these rats, several studies using in vivo and in vitro hyperinsulinemic euglycemic clamp techniques reported hepatic IR and a decrease of insulin sensitivity in extrahepatic tissues of GK rats^12^.

In women with PCOS, IR is a prevalent feature and, depending on the cohorts and the method used, it is encountered in 60–80% of these patients^14^. Since the sensitivity to insulin of both lean and obese women with PCOS is impaired, the task force of the Androgen Excess Society has recommended screening for impaired glucose tolerance (IGT) for all women with the PCOS^15^. In our work, following an intraperitoneal glucose injection (Fig. 1c), GK rats presented an alteration in the kinetics of plasma insulin at 5 months. Plasma insulin levels during the acute phase of insulin release were indeed lower in GK rats, whereas during the late phase of insulin release they were higher than in the Wistar controls (Fig. 1d). Moreover, in GK rats, the insulinogenic index (i.e., the mean incremental insulin and glucose area ratio) was lower than in controls (Wistar: 0.03 ± 0.009; GK: 0.002 ± 0.0006; *p* < 0.001). This might result from the defective β-cell function observed in GK rats, which is also present in some women with PCOS and in daughters of women with PCOS around the time of peri-puberty^16^.

### Dyslipidemia in GK rats

As the prevalence of dyslipidemia is three times higher in patients with PCOS compared to control individuals, we then measured serum lipids and lipoprotein concentrations in 3 and 6 month-old GK rats. Non-esterified fatty acids (NEFA) and triacylglycerol (TG) levels were increased in GK rats at both ages (Fig. 1e, f). Similarly, a lipidomic study indicated that NEFA and TG serum concentrations are higher in both lean and obese women with PCOS than in healthy women^17^. In women with PCOS, IR was shown to impair both lipid oxidation and the insulin-mediated suppression of lipolysis^18^. Likewise, a loss of the anti-lipolytic effect of insulin could explain the high NEFA levels in GK rats.

There was no evidence for a difference of the high-density lipoprotein-cholesterol (HDL-C) concentration between GK rats and controls at 3 months, but it was higher in GK rats at 6 months (Fig. 1g). Furthermore, there was a rise in the low-density lipoprotein-cholesterol (LDL-C) concentration in GK rats at both 3 and 6 months (Fig. 1h). Clinical investigations have shown that LDL-C plasma level increases by around 30% in women with PCOS independently of their BMI, but it is not affected in the context of other IR states^19^. These high LDL-C levels were also reported in the mothers and in the descendants of women with PCOS^20^. There is no indication that IR is the primary driver of dyslipidemia neither in women with PCOS as suggested by the absence of effect of insulin sensitizing drugs^19^, nor in GK rats since they develop dyslipidemia prior to presenting IR^12^. The reason for the increase of LDL-C levels in women with PCOS is not yet clear but could be related to hyperandrogenism^21^.

### Excess of adiposity in GK rats

**Fig. 2:**
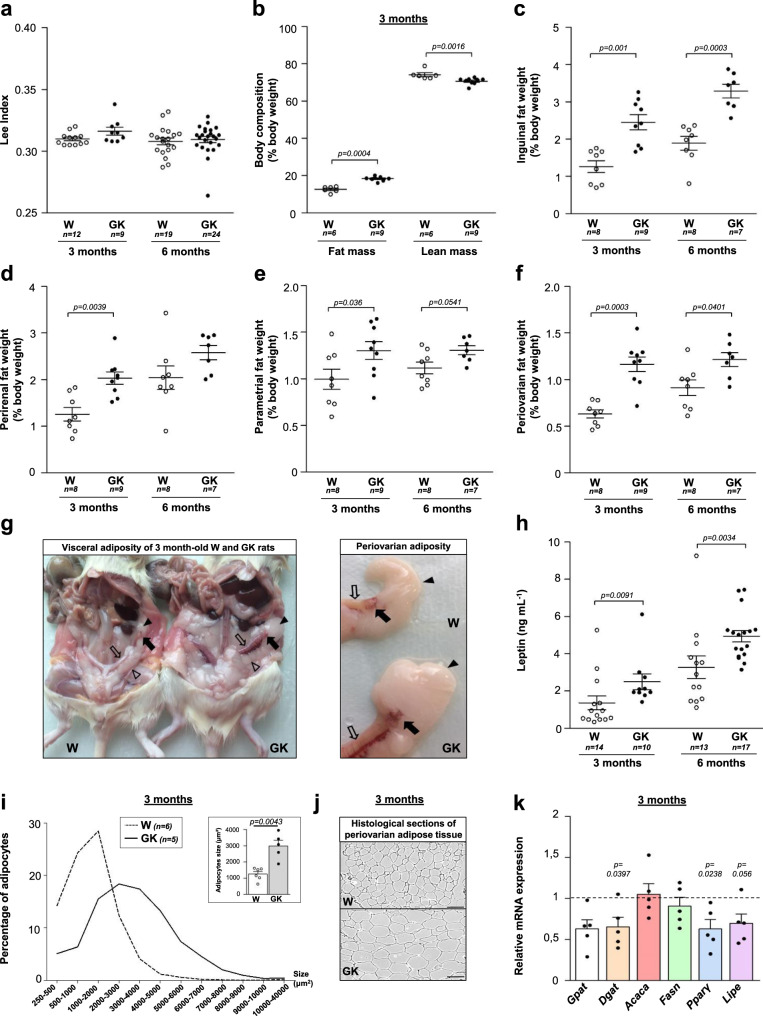
Adiposity of Wistar and GK rats. **a** Lee index of 3 and 6 month-old Wistar (W, open symbols) and GK (closed symbols) rats. **b** Amounts of fat mass and lean mass expressed as a percentage of body weight of 3 month-old Wistar and GK rats. **c**–**f** Weight of inguinal (**c**), perirenal (**d**), parametrial (**e**), and periovarian (**f**) fat depots expressed as the percentage of the body weight in 3 and 6 month-old Wistar and GK rats. **g** Representative abdominal cavity (left panel) and periovarian and parametrial fat (right panel) of Wistar (*n* = 8) and GK (*n* = 9) rats at 3 months. Black arrow: ovary; white arrow: oviduct; black arrowhead: periovarian fat; white arrowhead: parametrial fat. **h** Assessment of serum leptin levels in 3 and 6 month-old Wistar and GK rats. **i** Adipocytes size measurement on histological sections (**j**) from 3 month-old Wistar (dotted line) and GK (full line) rat periovarian adipose tissue, expressed as percentage of adipocytes in each size class. Statistical differences in adipocyte surface distribution between GK and Wistar rats were analyzed by *χ*^2^ (*p* < 0.0001). The inset in (**i**) shows the mean ± SEM adipocyte surface in Wistar and GK rats. **j** Representative images of 3 month-old Wistar (*n* = 6) and GK (*n* = 5) rats periovarian adipose tissue sections (Scale bar = 100 µm). **k** Expression of marker genes of triglyceride esterification (*Gpat* (white)*, Dgat* (peach), lipogenesis (*Acaca* (salmon)*, Fasn* (green)), adipocyte differentiation (*Pparγ* (blue)) and lipolysis (*Lipe* (purple)) in 3 month-old GK rats periovarian adipose tissue, expressed as box with scatter blot with mean ± SEM (*n* = 5). Unless indicated, data are represented as scatter blot with mean ± SEM. *p*-values are from two-sided Mann–Whitney *U* test for unpaired comparisons (GK vs. Wistar rats). *n* represents the number of biologically independent animals in each group. Source data are provided as a Source Data file.

To further characterize the adiposity of GK rats, we then measured their Lee index (∛body weight per size (g cm^−1^) (Fig. 2a). At the same age, it was comparable in GK and control rats, excluding the confounding influence of obesity. In line with previous reports^22^, the fat mass of GK rats was ~37% higher than that of controls and conversely, the lean mass was reduced by 5% at 3 months (Fig. 2b), similar to that observed in lean patients with PCOS^23^.

At 3 and 6 months, adipose tissue mass (inguinal, perirenal, and parametrial) was higher in GK rats than in Wistar rats (Fig. 2c–e) demonstrating an expansion in adiposity. This finding is in line with observations in testosterone-induced models of PCOS or in patients with the metabolic syndrome^9^. Non-obese patients with PCOS display visceral accumulation of fat, which could contribute to the development of disorders in glucose and lipid metabolism^24^. GK rats presented hypertrophic periovarian fat (Fig. 2f–g), which almost doubled compared to controls at 3 months (Fig. 2f). Periovarian fat was recently suggested to regulate ovarian folliculogenesis^25^. Indeed, the surgical removal of periovarian fat pad in mice leads to a delayed antral follicles development. As observed for both lean and obese women with PCOS^26^, GK rats exhibited a hyperleptinemic state consistent with their increased adiposity (Fig. 2h).

We then focused on periovarian adipose tissue and we observed that this VAT contained enlarged adipocytes in GK rats at 3 months, with a mean area 2.4 fold-higher than controls, as previously reported^22^ (Fig. 2i, j). The sub-cutaneous adipose tissue (SCAT) of women with PCOS also exhibits enlarged adipocytes even in the absence of obesity^27^. However, SCAT has a limited capacity to increase its mass safely. When it reaches its maximal expandability capacity, lipids may spill over in non-SCAT, such as VAT, liver, and skeletal muscle. The expandability concept implies a “metabolic setpoint” which could be dependent on high concentrations of insulin and androgens^28^. Indeed, accumulating evidences suggest that androgens are involved in the modification of adipose tissue cellularity. Since adipocyte size reflects the balance between triglyceride storage and mobilization, in an attempt to identify the mechanisms underlying adipocyte hypertrophy in periovarian adipose tissue, we determined the mRNA expression levels of the key enzyme of lipolysis (hormone sensitive type, *Lipe*), and of target genes involved in lipogenesis (acetyl-CoA carboxylase alpha, *Acaca* and fatty acid synthase, *Fasn*) and in fatty-acid esterification (glycerol-3-phosphate acyltransferase, *Gpat* and diacylglycerol O-acyltransferase 1, *Dgat*) in GK and Wistar rats. In GK rats, there was no evidence for a modification of lipogenesis gene expression while the level of *Dgat* expression decreased (Fig. 2k), consistent with the known inhibitory effect of androgens on *Dgat* in human adipocytes cultures^29^. A decreasing tendency of *Lipe* expression was observed in GK rats, suggesting that an altered lipolysis could contribute to the adipocyte hypertrophy in these animals. In women with PCOS, detailed molecular investigations of lipid metabolism are limited. However, Ek et al. ^30^ found that catecholamine-induced adipocyte lipolysis was increased in VAT from women with PCOS, although expression of the two isoforms of hormone-sensitive type were either not modified or decreased.

We also investigated the expression of *PPARγ*, the master adipogenic transcription factor responsible for adipogenic conversion. *PPARγ* mRNA levels were significantly reduced by 33% in GK rats (Fig. 2k). In many species, including human, androgens do not modify preadipocyte proliferation but they do inhibit their differentiation^6^. In particular, Chazenbalk et al. showed that they inhibit human multipotent adipose stem cells differentiation and *PPARγ* mRNA expression in vitro^31^.

Taken together, our results suggest that despite an alteration of periovarian adipocyte conversion into mature adipocytes due to the decrease of *PPARγ* expression, the expansion in periovarian adipocyte tissue mass could be due to reduced lipolysis and resulting adipocyte hypertrophy. Further investigations will be required to assess the molecular mechanisms underlying the changes in periovarian adipocyte maturation and size.

### Alteration of ovarian functions in GK rats

**Fig. 3:**
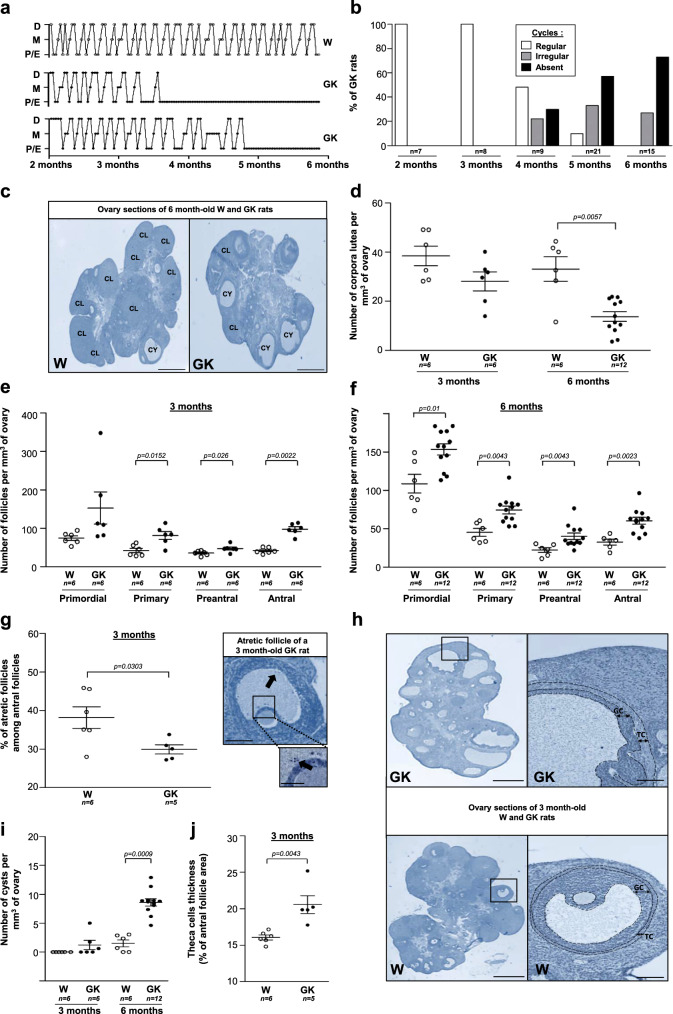
Estrous cycle profile and ovarian phenotype of Wistar and GK rats. **a** Examples of estrous cycle profile of one Wistar (W) and two GK rats, showing the evolution of the cycle stage (Proestrus–Estrus P–E, Metestrus M, Diestrus D) according to the age of the rat. **b** Percentage of GK rats with regular, irregular cycles or an absence of cycle at 2, 3, 4, 5, and 6 months. **c** Representative hematoxylin–eosin-stained ovaries sections of 6-month-old Wistar (left, *n* = 6) and GK (right, *n* = 12) rats. CL corpora lutea, CY cyst. Scale bar = 1 mm. **d** Number of corpora lutea per mm^3^ of ovary assessed in 3 and 6 month-old Wistar (W, open symbols) and GK (closed symbols) rats. **e**, **f** Number of primordial, primary, preantral, and antral follicles per mm^3^ of ovary counted on 3 (**e**) and 6 (**f**) month-old Wistar and GK rat ovarian sections. **g** Percentage of atretic antral follicles, characterized by several detached pycnotic granulosa cells (arrow) in the antrum, determined in 3-month-old Wistar and GK rat ovaries. Scale bars = 100 and 25 µm. **h** Representative hematoxylin–eosin stained ovary sections of 3-month-old GK (top, *n* = 5) and Wistar (bottom, *n* = 6) rats (left). Scale bar = 1 mm. High magnifications of the boxes areas display a GK rat cyst wall (top) and a Wistar (bottom) rat antral follicle (right). GC granulosa cells, TC theca cells. Scale bar = 100 µm. **i** Number of cysts per mm^3^ of ovary determined in 3 and 6 month-old Wistar and GK ovaries. **j** Mean value of the theca cells layer thickness measured on antral follicles in 3 month-old Wistar and GK rat ovaries, expressed as a percentage of the follicle area. Results were analyzed by two-sided Mann–Whitney *U* test for unpaired comparisons (GK vs. Wistar rats). Data are represented as scatter blot with mean ± SEM. *n* represents the number of biologically independent animals in each group. Source data are provided as a Source Data file.

As oligo/anovulation is a pivotal feature of PCOS and the primary reason for consultation in assisted reproductive technology (ART) centers, we first monitored the periodicity of ovarian cycles by analyzing vaginal smears in Wistar and GK rats (Fig. 3a, b). The daily analysis of vaginal smears over 6 consecutive months (Fig. 3b) revealed that, at 2 and 3 months, 100% of GK rats displayed normal estrous cyclicity with the presence of all stages of the estrous cycle and a normal cycle length. Between 3.5 and 5 months, the percentage of GK rats with longer cycles progressively increased and at 6 months, 75% of GK rats were acyclic. Pinto-Souza et al. only showed a delay from proestrus to estrus at 6 months, probably because of the small number of animals^13^. The estrous cycle of GK rats was blocked at the proestrus–estrus stages and the metestrus and diestrus stages were entirely absent (Fig. 3a). In contrast, control Wistar rats displayed a normal cycle length with the presence of all stages of the estrous cycle over the span of a 6-month period. Consistent with this oligo/anovulatory phenotype, the ovarian histology of GK rats presented fewer corpora lutea at 3 and 6 months (Fig. 3c, d). Similarly, women with PCOS become infertile progressively, with 70% of oligo/amenorrhea. Conversely, ~90–95% of anovulatory women have the PCOS, which explains why this trait stands as one of the PCOS Rotterdam diagnosis criteria. Moreover, since the accumulation of antral follicles represents another Rotterdam diagnosis criterion, the follicle content of GK rat ovaries was then analyzed. To determine which specific stages of follicular development were selectively affected, follicles were divided into four different classes: primordial, primary, preantral, and antral follicles. As early as 3 months of age, the ovaries of the GK rats exhibited an increase in the number of antral follicles and in the number of primary and preantral follicles as well (Fig. 3e, f). Similarly, there is evidence that the pre-antral stages of folliculogenesis are also altered in women with PCOS, since the number of their primary and secondary follicles are roughly twice the number of those observed in a normal ovary^32^.

As some authors suggested that the increase in the number of follicles could be partly explained by a reduction of the rate of follicle atresia in women with PCOS^33^, the next step was to determine the number of atretic follicles in GK rats. In keeping with this hypothesis, a decreased number of atretic follicles, characterized by several detached pycnotic granulosa cells (GC) in the antrum, disorganized GC, and a degenerating oocyte was observed (Fig. 3g). The ovarian morphologic changes of GK rats included an increased incidence of cystic follicles (Fig. 3i, h) defined as dilated follicles containing cavities filled with follicular fluid, and lined with one to five cell layers thick of round-to-flattened GC. The presence of some cysts in Wistar controls at 6 months was also observed, probably due to the spontaneous formation of cysts during reproductive aging^34^. As with women with PCOS, the cysts in GK rats were typically located in the periphery of the ovary (Fig. 3h, top). Furthermore, in agreement with the typical ovarian morphological alterations observed in patients with PCOS and in androgen-treated models of PCOS, the GK rat cystic follicles presented a thin layer of GC (Fig. 3h) and a thickened theca cell-layer area (Fig. 3j, h). Interestingly, insulin also enhances ovarian growth and follicular cyst formation in rats^35^ and stimulates human theca cell proliferation^36^.

### Reproductive endocrine disorders in GK rats

**Fig. 4:**
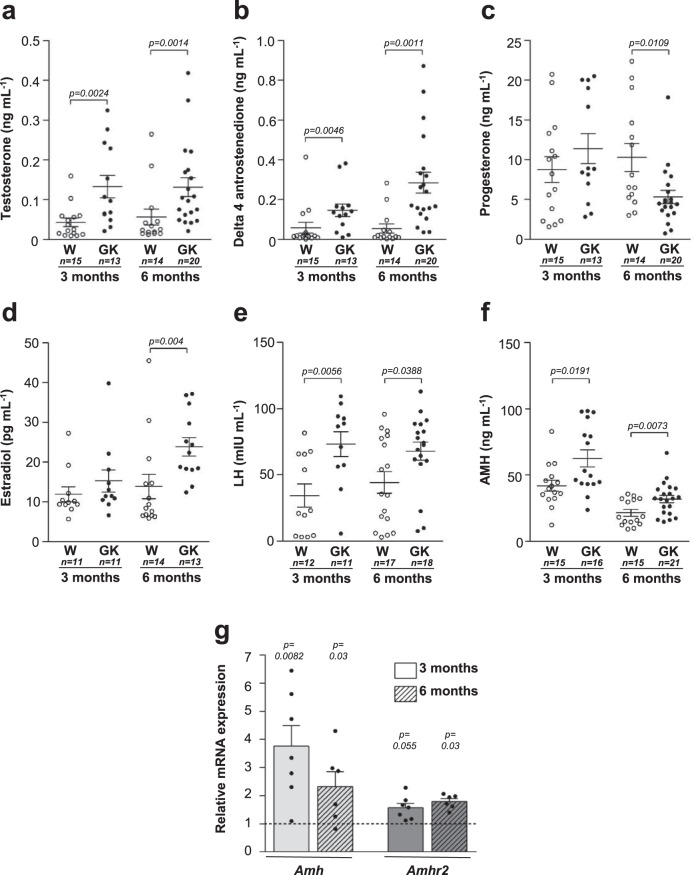
Hormonal profile of Wistar and GK rats. **a**–**f** Serum levels of Testosterone (**a**), Delta-4 androstenedione (**b**), Progesterone (**c**), Estradiol (**d**), Luteinizing hormone (LH) (**e**), and anti-Müllerian Hormone (AMH) (**f**) of 3 and 6 month-old Wistar (W, open symbols) and GK (closed symbols) rats. **g** *Amh* (light gray) and *Amhr2* (dark gray) mRNA levels in 3 (non-hatched) and 6 (hatched) month-old GK rat ovaries, expressed as box with scatter blot with mean ± SEM (3 months GK rat: *n* = 7; 6 month-old GK rats: *n* = 6, fold change relative to Wistar rats; 3 months Wistar rats: *n* = 6; 6 month-old Wistar rats: *n* = 5). Unless indicated, data are represented as scatter blot with mean ± SEM. *p*-values are from two-sided Mann–Whitney *U* test for unpaired comparisons (GK vs. Wistar rats). *n* represents the number of biologically independent animals in each group. Source data are provided as a Source Data file.

Since hyperandrogenism, another of the Rotterdam criteria, is considered to be a mandatory feature of the PCOS, serum testosterone and delta4-androstenedione levels were measured in GK and Wistar rats using the liquid chromatography mass spectrometry (LC–MS/MS) approach^37^. Despite the variable levels observed in GK rats, circulating testosterone and delta4-androstenedione levels were markedly elevated in both ovulatory 3-month-old GK rats and in anovulatory 6-month-old GK rats compared to age-matched Wistar rats (Fig. 4a, b). Similarly, theca cell steroidogenesis is abnormal in both ovulatory and anovulatory subjects in women with PCOS. The increase of delta4-androstenedione—a precursor of testosterone—in GK rats suggests a global up-regulation of steroidogenic enzymes as observed in theca cells from women with PCOS^9^. The rise of androgen levels in GK rats may be related to their high levels of NEFA, which stimulate androgen production in both rats and women^38, 39^. In addition, this result fits with previous studies demonstrating that insulin stimulates ovarian androgen production and reduces hepatic sex hormone-binding globulin synthesis in various species, thereby increasing the levels of total and bioavailable androgens (reviewed in ref. ^40^). In turn, this hyperandrogenism is likely involved in abdominal fat accumulation in women with PCOS as suggested by the decrease of their visceral fat mass when they were treated with androgen receptor antagonists^21^.

To further investigate the ovulation defects observed in GK rats, we also measured progesterone levels by mass spectrometry (Fig. 4c). At 3 months, there was no evidence for a difference between the progesterone levels of GK rats and Wistar controls, whereas at 6 months progesterone levels decreased in GK rats, which is consistent with their oligo/anovulation. There was no evidence for a difference in the estradiol levels between GK and Wistar rats at 3 months, whereas there were increased in anovulatory GK rats at 6 months (Fig. 4d), in line with the increase of estradiol levels observed in anovulatory women with PCOS^41^.

Because 60% of women with PCOS present a three-fold increase in LH levels (The Rotterdam ESHRE/ASRM sponsored PCOS), and since LH acts synergistically with insulin to enhance theca cell androgen production^9^, we measured LH concentration in GK rats. At both 3 and 6 months, we found a mild elevation of LH in GK rats as compared to Wistar controls (Fig. 4e), consistent with the fact that not all women with PCOS have increased serum LH levels and/or an elevated LH to FSH ratio^42^. LH is not sufficient on its own to produce a theca cell hyperplasia because no thickened theca tunica has been observed in the ovaries of mice overexpressing the β subunit of LH^4^. Theca cell hyperplasia is likely also due to hyperandrogenism and hyperinsulinemia.

Lastly, since AMH levels are two–three-fold higher in women with PCOS^43^ and regulate LH pulsatility^44^, we also measured serum AMH levels in GK rats and Wistar controls. We showed that, at 3 months of age, there were higher serum AMH levels in GK rats (Fig. 4f). Moreover, the expression of *Amh* and of its specific receptor (*Amhr2*) mRNAs was up-regulated in the ovaries of GK rats as compared to Wistar controls (Fig. 4g). This suggests that, as observed in women with PCOS^45^, the rise of AMH levels in GK rats results from both the increased number of follicles and the enhancement of AMH production per GC. We have recently shown that the overexpression of *AMH* and *AMHR2* in GC from women with PCOS is due to a dysregulation by androgens, estradiol, and LH^46, 47^. In addition, insulin might also contribute to an elevated AMH production in women with PCOS as suggested by the positive correlation between fasting insulin levels and AMH concentration in women with PCOS^48^, and by the stimulatory effect of insulin on AMH expression in human luteinized GC^49^.

### Disorders of puberty in female GK rat offspring

**Fig. 5:**
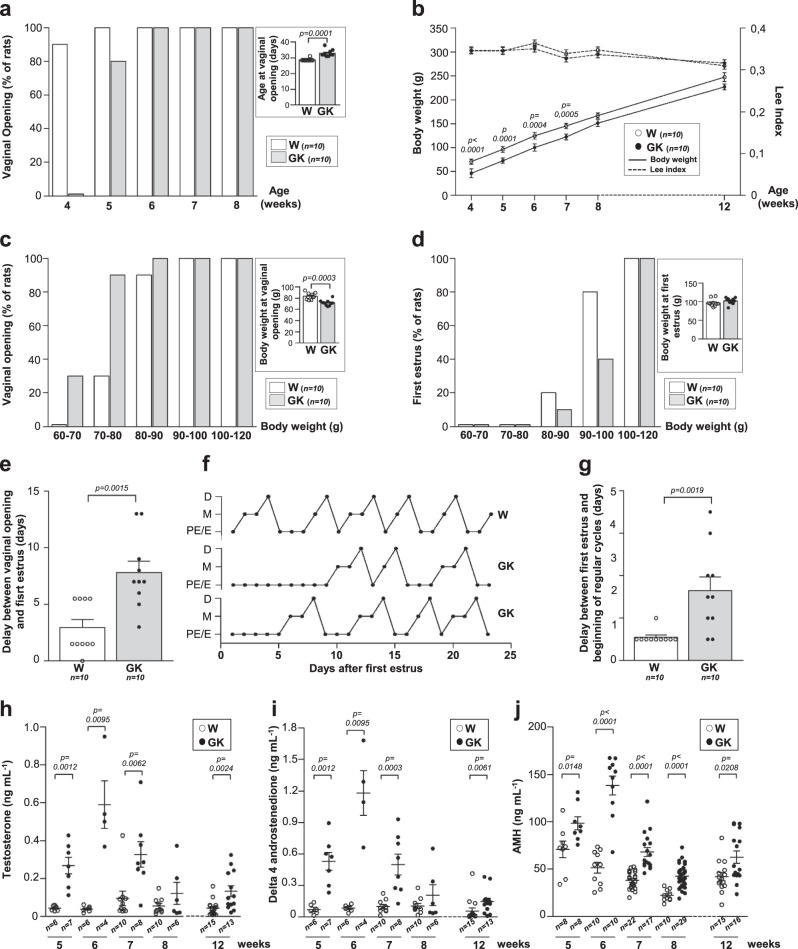
Study of pubertal development of Wistar and GK rats. **a** Percentage of rats with opened vaginas from 4 to 8 weeks of age. Inset shows the mean age of the rats at the vaginal opening. **b** Evolution of body weight (full line) and of Lee index (dotted line) of Wistar and GK rats between 4 and 12 weeks. *p*-values are from two-sided Mann–Whitney *U* tests followed by a post-hoc Bonferroni’s adjustment. **c** Percentage of rats with vaginal opening in each body weight class. Inset shows the mean weight of rats at vaginal opening. **d** Percentage of rats at first estrus for each body weight class. Inset shows the mean weight of rats at the time of the first estrus. **e** Delay between vaginal opening and the first estrus. **f** Illustration of estrous cycle profiles from the first estrus for 1 Wistar and 2 GK rats. Proestrus–Estrus P–E, Metestrus M, Diestrus D. **g** Delay between first estrus and the beginning of regular estrous cycles in Wistar and GK rats. **h**–**j** Serum levels of Testosterone (**h**), Delta-4-androstenedione (**i**), and AMH (**j**) assessed on 5–12-week-old Wistar and GK rats. Data are represented as box with scatter blot with mean ± SEM (**a**, **c**, **d** insets and **e**, **g**), as mean ± SEM (**b**) or as scatter blot with mean ± SEM (**h**–**j**). *p*-values are from two-sided Mann–Whitney *U* test for unpaired comparisons (GK vs. Wistar rats). *n* represents the number of biologically independent animals in each group. Wistar: white, GK: gray. Source data are provided as a Source Data file.

Since ~50% of the daughters of women with PCOS are more prone to develop PCOS phenotype at puberty^50^, we studied different puberty parameters in the daughters of adult GK and Wistar rats. The GK and Wistar female offspring F1 were monitored daily for vaginal opening, the first marker of puberty, between 4 and 8 weeks after birth. In Wistar rats, 100% had opened vagina at 5 weeks whereas the onset of vaginal opening occurred later in GK rats in which the puberty was reached for 100% of them at 6 weeks (Fig. 5a). However, the body weight of GK rats was significantly decreased compared to Wistar rats during the puberty period (Fig. 5b). In both animals and humans, the age of puberty appears to be more related to body weight than to chronologic age. Consequently, we have normalized the onset of puberty in GK and Wistar rat offspring to the body weight. In this case, vaginal opening occurred at a lower body weight in GK rats (Fig. 5c), reflecting a precocious puberty, which is consistent with studies in adolescents with PCOS^51, 52^. Indeed, pubarche, which refers to the first appearance of pubic hair at puberty, appears to be earlier in adolescents with PCOS^51^. However, the age at menarche, corresponding to the first menstrual bleeding seems to be earlier in overweight and obese patients with PCOS and later in lean patients with PCOS. The first estrus was also delayed in GK rats compared to Wistar controls (Fig. 5d, e and Supplementary Fig. 1a), which is consistent with this model being representative of the lean PCOS phenotype. In addition, 60% of GK rat offspring failed to reach regular estrous cyclicity as assessed by the lack of ovulation and irregular estrous cycles during several days after the first estrus (Fig. 5f). Furthermore, the delay between first estrus appearance and the establishment of regular estrous cycle was also significantly higher in GK rats compared to control Wistar rats (Fig. 5g). In human, Wiksten-Almströmer et al. ^53^ showed that after 6 years of follow-up, 59% of teenagers who have irregular menstrual cycles fulfill the PCOS criteria. Persistent oligomenorrhea was suggested as a requirement for the diagnosis of PCOS in adolescents by Endocrine Society’s PCOS clinical practice guidelines^15^.

The other prevalent feature in adolescents with PCOS is hyperandrogenism^15^. Therefore, we measured testosterone and delta4-andostenedione levels by mass spectrometry between 5 and 12 weeks of age (Fig. 5h, i) and we observed higher testosterone and delta4-andostenedione levels in GK rats offspring compared to Wistar controls, which is consistent with the hyperandrogenism observed in adolescents with PCOS and in the daughters of women with PCOS^54^. Because postpubertal girls with a history of premature pubarche have high ovarian androgens levels (45% vs. 3% in the normal population)^55^, the premature vaginal opening observed in GK rats may be associated with their hyperandrogenism.

Finally, since AMH levels are significantly elevated in adolescents with PCOS^56^ and in daughters of women with PCOS who have an increased risk to develop a PCOS^57^, we measured serum AMH levels in GK and Wistar rat offspring by longitudinal evaluation. In Wistar rats, circulating AMH levels fell by around 50% between 5 and 8 weeks (Fig. 5j) which is similar to the 30% reduction in AMH levels during the first 2 years after pubertal onset observed in healthy adolescents^58^. At all ages, GK rat offspring exhibited a significant increase in serum AMH levels compared to Wistar controls (Fig. 5j), reflecting an increased follicle pool. In addition, the peak of AMH levels at 6 weeks in GK rats coincided with the peak of androgens and with the high glucose (Supplementary Fig. 1b) and insulin^12^ levels. Indeed, the GK rats already exhibit the 3-month-old metabolic phenotype at 6 weeks of age, similarly to what is observed in adolescents with PCOS^59^.

Firstly, our results as a whole placed the origin of the GK phenotype before puberty and, secondly, the fact that female GK offspring display the major cardinal features of adolescents with PCOS suggests a spontaneous transgenerational inheritance of the PCOS phenotype in GK rats.

### Alteration of maternal–fetal environment in GK rats

**Fig. 6:**
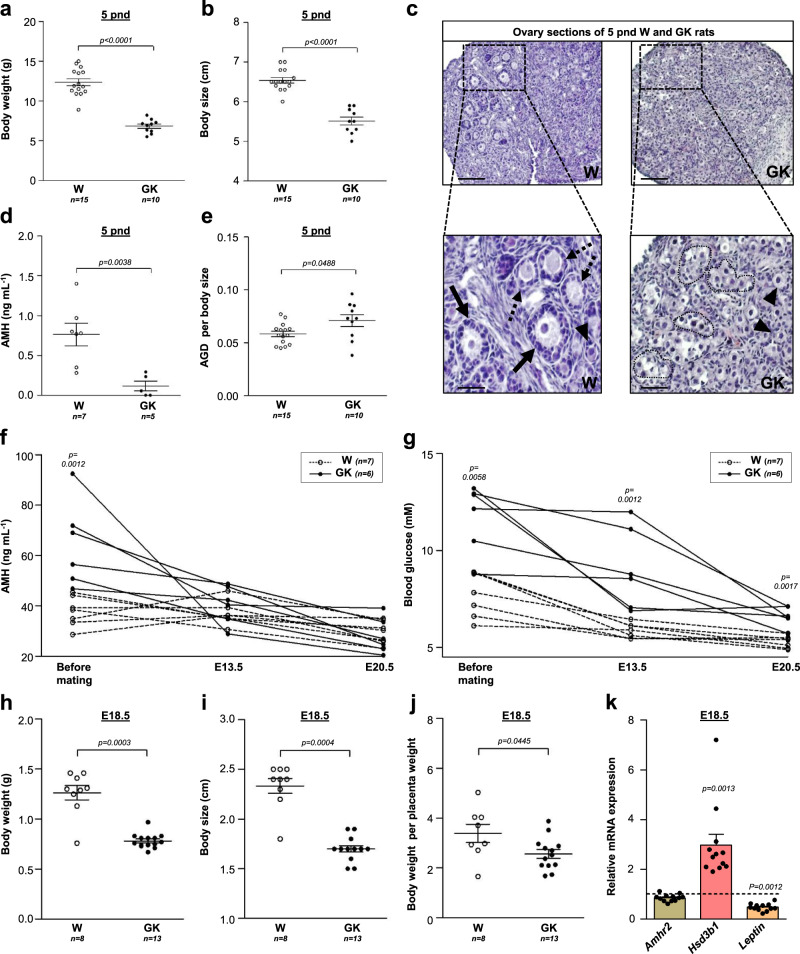
Study of fetal and postnatal development of Wistar and GK rats. **a**, **b** Body weight (**a**) and size (**b**) of 5 postnatal days (pnd) Wistar (W, open symbols) and GK (closed symbols) rats. **c** Representative histological sections of hematoxylin–eosin-stained ovaries of 5 pnd Wistar (left, *n* = 7) and GK (right, *n* = 5) rats. Black arrows: secondary follicles; dotted arrows: primary follicles; arrowheads: primordial follicles. Black dotted lines encircle the oocyte nest. Scale bars = 100 or 25 µm. **d** Serum AMH levels assessed on 5 pnd rats. **e** Anogenital distance (AGD) of 5 pnd rats. **f**, **g** Serum AMH (**f**) and blood glucose (**g**) levels measured before mating and at embryonic days (E) 13.5 and E20.5 of gestation on 2 month-old Wistar and GK rats. **h**, **i** Body weight (**h**) and size (**i**) of Wistar and GK rats fetuses at E18.5. **j** Ratio between body weight and placenta weight of E18.5 Wistar and GK rat fetuses. **k** *Amhr2* (beige)*, Hsd3b1* (salmon), and *Leptin* (orange) mRNA levels in placenta of E18.5 GK rat fetuses, expressed as box with scatter blot with mean ± SEM (*GK: n* = 12; fold change relative to Wistar rats; W: *n* = 9). Unless indicated, data are represented as scatter blot with mean ± SEM. *p*-values are from two-sided Mann–Whitney *U* test for unpaired comparisons (GK vs. Wistar rats). *n* represents the number of biologically independent animals in each group. Source data are provided as a Source Data file.

We then studied the daughters of adult GK rats when they in turn became adults (F1) in order to determine the origin of the GK phenotype in their offspring (F2). Since post-natal consequences of in utero PCOS maternal environment can be visible in the newborn^6^, we first carried out an anthropometric analysis of the female offspring at 5 post-natal days (pnd). Consistent with previous reports in women with PCOS and in androgenized or diabetic animal models^6^, F2 GK rats exhibited low birth weight and were smaller than Wistar controls (Fig. 6a, b), suggesting intra-uterine growth retardation. In line with these results, the development of GK ovaries was delayed in comparison to that of Wistar rats. In fact, 5 pnd GK ovaries presented oocyte nest but were devoided of primary follicles whereas primordial, primary and secondary follicles were present in Wistar rat ovaries (Fig. 6c). In keeping with this delayed follicular development, we detected a significant decrease of serum AMH levels in GK in comparison to Wistar rats at 5 pnd (Fig. 6d). We did not measure steroids because following the neonatal testosterone surge observed in both males and females, circulating testosterone remains low up to the onset of puberty. However, compared to control Wistar offspring, the 5 pnd GK female offspring displayed significantly elongated anogenital distance (AGD) (Fig. 6e), a reliable marker of gestational fetal androgenic exposure. Similarly, several studies have shown that AGD is longer in women with PCOS and in the newborn daughters of these women^6^. This in utero hyperandrogenism in GK rats may contribute to their pancreatic β-cell dysfunction observed as early as at embryonic day (E) 16.5^12^. Since recent findings reported that high gestational AMH exposure during pregnancy contributes to in utero hyperandrogenism^7^, we then undertook to determine the longitudinal profile of AMH during pregnancy in 6 F1 GK and 6 Wistar rats (Fig. 6f). In both groups, the serum AMH levels declined during gestation (mean decrease of 22% in Wistar rats and 48.5% in GK rats) at 20.5 days of gestation compared to AMH values before gestation. In addition, after 13.5 days into the gestation, the median levels of AMH was higher in the GK group than in Wistar group (41.3 vs. 36.1 ng/ml, respectively) but there was no evidence for a significant difference between the two, probably as a result of the small number animals in each group and of the overlap in AMH levels between the GK and Wistar groups. However, there was no evidence for a difference between AMH levels from GK and Wistar F1 dams at term gestation (20.5 days of gestation) (Fig. 6f) as described in women with PCOS^60^. In contrast, Piltoneen et al. reported that pregnant women with PCOS present higher serum AMH levels than control women at term^61^. This discrepancy could be explained by the mixture of fetal gender influence on maternal AMH levels in rats because the levels of AMH were significantly higher in women with PCOS carrying female fetuses but this was not the case in women with PCOS carrying male fetuses^61^. In addition, since women with PCOS continue to present high serum glucose levels during pregnancy, we analyzed the longitudinal profile of glycemia in GK and Wistar F1 dams (Fig. 6g). In both groups, glycemia decreased as gestation advanced but remained higher in GK than in Wistar dams until term pregnancies. Then, to understand the maternal–fetal relationships, we studied in another set of experiments the placenta of F1 GK rats and their F2 female GK fetuses at E18.5. We found that there was a significant increase in the mean number of aborted embryos in three litters of GK compared to Wistar rats (3.6 ± 3.2 vs. 0), consistent with the higher proportion of miscarriage observed in women with PCOS^62^. In addition, confirming the results obtained at 5 pnd, female F2 GK fetuses exhibited low weight (Fig. 6h) and were smaller (Fig. 6i) than Wistar fetuses. Finally, since a large body of evidence implicates the placenta as a mediator of pregnancy complications, we determined the body (BW) to placental weight (PW) ratio which allows us to infer how effectively the placenta has adapted to fetal growth needs (Fig. 6j). The BW per PW ratio was significantly lower in GK vs. Wistar rats as previously described in non-obese women with PCOS^63^. We also examined the expression of several markers of placental function (Fig. 6k). We did not find evidence for differences in placenta *Amhr2* expression between GK and Wistar rats, as previously observed in AMH-induced PCOS mice^7^. In contrast, we found in GK rats an increase in mRNA levels of *Hsd3b1* (Fig. 6k), the enzyme responsible for progesterone synthesis, which is also over-expressed in the placenta of women with PCOS^64^. Lastly, since it has been suggested that the deregulation of leptin function in the placenta is implicated in the pathogenesis of various disorders during pregnancy, such as intra-uterine growth retardation and recurrent miscarriages^65^, we analyzed *Leptin* expression in the placenta at E18.5. We found that it was about 2-fold lower in GK rats compared to Wistar rats. However, further investigations are needed to confirm this finding and to understand the role/regulation of placental leptin.

Our findings are in keeping with the concept of DoHAD whereby in utero alterations, namely hyperandrogenism, hyperglycemia, and high levels of AMH in GK rats could cause placenta alterations, poor intra-uterine growth and the low birth weight of their offspring, which could lead to the reproductive and metabolic alterations observed during puberty and in adulthood.

### Ovarian response to gonadotropin stimulation of GK rats

**Fig. 7:**
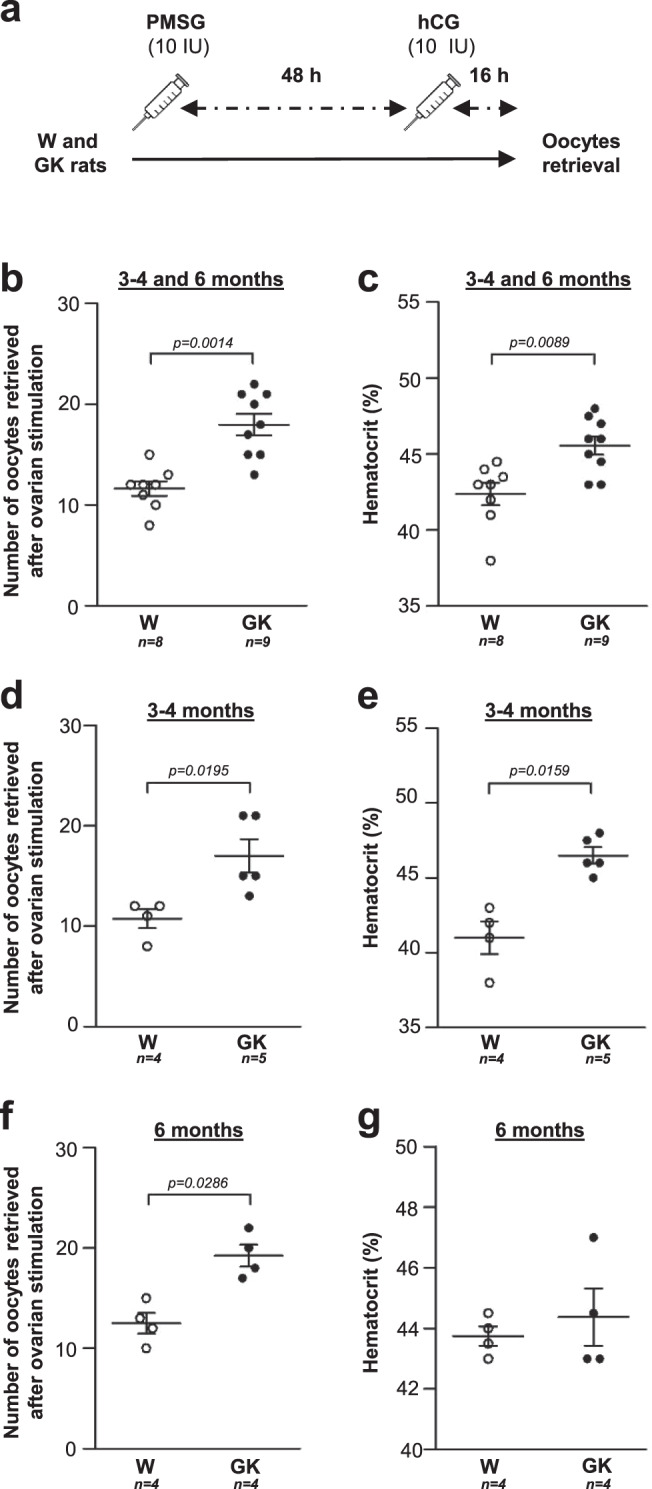
Ovarian gonadotropin stimulation of Wistar and GK rats. **a** 3–4 and 6 month-old Wistar (W, open symbols) and GK (closed symbols) rats were injected with 10 IU of PMSG followed 48 h later with 10 IU of hCG to induce ovulation. **b**, **d**, **f** Number of oocytes retrieved after ovarian stimulation in Wistar and GK rats. **c**, **e**, **g** Hematocrit in Wistar and GK rats. Panels **b**, **c** show the combined results for 3–4 and 6 month-old rats; and panels **d**, **e** and **f**, **g** represent results for 3–4 and 6 month-old rats, respectively. Data are represented as scatter blot with mean ± SEM. *p*-values are from two-sided Mann–Whitney *U* test for unpaired comparisons (GK vs. Wistar rats). *n* represents the number of biologically independent animals in each group. Source data are provided as a Source Data file.

Because adult GK rats present the three Rotterdam criteria, and ART and associated controlled ovarian stimulation are routinely used to treat the infertility of patients with PCOS, we then compared the number of oocytes retrieved in Wistar and GK rats after a classical gonadotropin stimulation of folliculogenesis and ovulation protocol. Consistent with an increased number of follicles in GK ovaries, treatment with 10 IU of pregnant mare serum gonadotropin (PMSG) followed after 48 h by a 10 IU human chorionic gonadotropin (hCG) injection (Fig. 7a), resulted 16 h later in a 1.5-fold higher number of oocytes in the ampulla of GK rats compared to Wistar controls (18 ± 1.1 vs. 11.6 ± 0.7) (Fig. 6b). This higher number of retrieved oocytes was observed separately at 3 months (17 ± 3.7 vs. 10.8 ± 1.9) (Fig. 7d) but also at 6 months in sterile GK rats (19.3 ± 2.2 vs. 12.5 ± 2.1) (Fig. 7f), suggesting that like in anovulatory women with PCOS, gonadotropin ovarian stimulation unblocks the maturation of their follicle pool. Finally, because patients with PCOS, in particular those presenting the three Rotterdam criteria, have a higher risk of developing an OHSS, the main iatrogenic complication of PCOS that can be aggravated by pregnancy^66^, we also measured rat hematocrit the day of oocyte retrieval. Indeed, hemoconcentration is one of symptoms of OHSS and hematocrit was shown recently to predict OHSS the day of ovarian puncture^67^. Hematocrit was found higher in compiled 3–4 and 6-month-old GK rats than in Wistar rats (Fig. 7c). We found that hematocrit was increased in GK compared to Wistar rats at 3–4 months (46.5 ± 1.2 vs. 41 ± 2.2) but there was no evidence for such a difference at 6 months (44.4 ± 1.9 vs. 43.8 ± 0.7) (Fig. 7c, e), which is consistent with a young age being a risk factor for developing an OHSS^68^.

Thus, in keeping with the higher incidence of OHSS among patients with PCOS, GK rats also present several OHSS features.

### Comparison between GK rats and PCOS women parameters

**Fig. 8:**
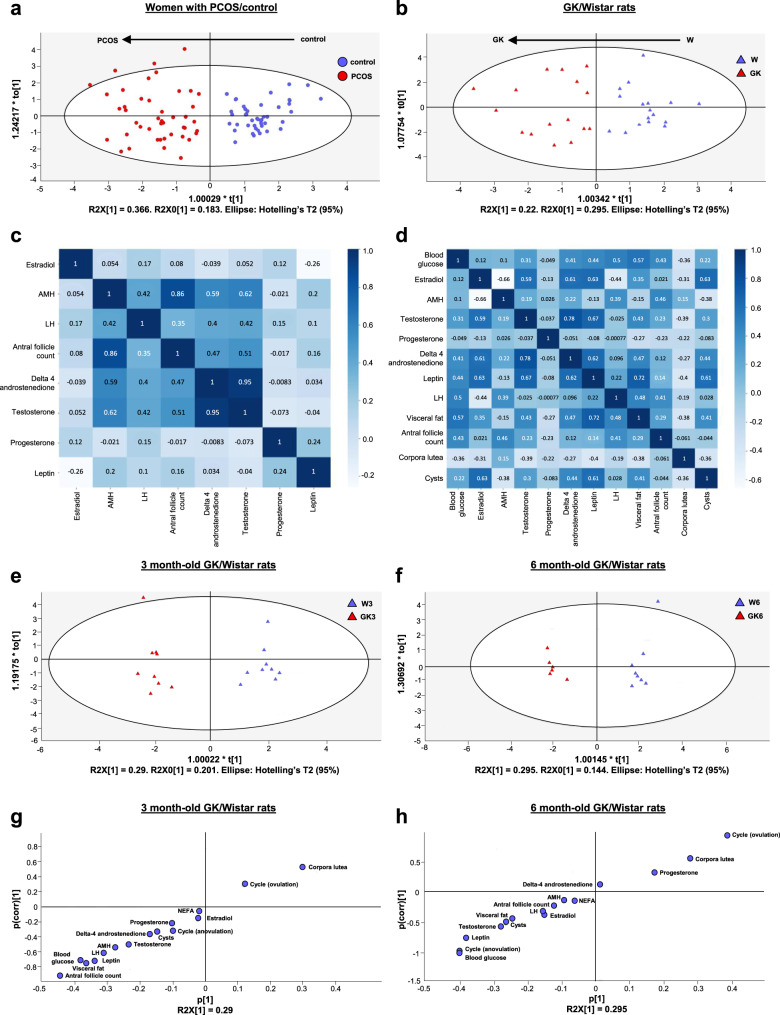
Multivariate regression analysis in women with PCOS and GK rats. **a** Orthogonal partial least-squares discriminant analysis (OPLS-DA) score plot for women with PCOS (*n* = 45, red dots) and control women (*n* = 44, blue dots) based on seven reproductive criteria (Supplementary Fig. 2a and Supplementary Table 1). The OPLS-DA score plot comprised one orthogonal and one predictive components, R2X(cum) = 0.549, R2Y(cum) = 0.810, Q2(cum) = 0.765. **b** OPLS-DA score plots for GK (*n* = 16, red triangles) and Wistar (*n* = 17, blue triangles) rats based on 11 reproductive criteria (Supplementary Fig. 2b). The OPLS-DA score plot comprised three orthogonal and one predictive components, R2X(cum) = 0.697, R2Y(cum) = 0.962, Q2(cum) = 0.871. **c**, **d** Pearson’s correlation matrix between reproductive and metabolic parameters for women (**c**) and rats (**d**). The stronger the correlation, the darker the color of blue. **e**, **f** OPLS-DA score plot based on 15 metabolic and reproductive criteria (Supplementary Fig. 2c, d) for 3 months GK (*n* = 9, red triangles) and Wistar rats (*n* = 9, blue triangles) (**e**) and for 6 months GK (*n* = 6, red triangles) and Wistar rats (*n* = 8, blue triangles) (**f**). The OPLS-DA score plot comprised three orthogonal and one predictive components; at 3 months: R2X(cum) = 0.697, R2Y(cum) = 0.962, Q2(cum) = 0.871 and at 6 months. R2X(cum) = 0.439, R2Y(cum) = 0.950, Q2(cum) = 0.702. **g**, **h** S-plot obtained from the OPLS-DA regression at 3 months (**g**) and 6 months (**h**) to identify the contribution of the 15 variables in the model (Pareto scaled data set). *n* represents the number of biologically independent women or animals in each group. Source data are provided as a Source Data file.

Finally, we sought to compare the major PCOS traits between GK rats and a cohort of lean women with PCOS displaying the three Rotterdam criteria (Supplementary Table 1). First, a multivariate model (PLS approach) was built to compare the cohort of patients with PCOS with control women. The parameters included in the model were the antral follicle count (AFC), serum AMH, LH and estradiol, and intrafollicular levels of testosterone, delta-4 androstenedione and progesterone (Supplementary Fig. 2a). As expected, the cluster of women with PCOS segregated from that of control women (Fig. 8a). Using this statistical approach with the same reproductive criteria, GK rats segregated from the Wistar rats (Fig. 8b, Supplementary Fig. 2b). Moreover, using a Pearson’s test, a positive correlation was observed between serum AMH levels and AFC or testosterone in GK rats as observed in women with PCOS^48^ (Fig. 8c–f). Thanks to this modeling approach, we were thus able to segregate GK rats from Wistar rats based on PCOS reproductive criteria without the metabolic key parameters at the origin of the GK strain.

In order to evaluate the impact of age on the relative contribution of metabolic and reproductive criteria in GK phenotype, we then distinguished the 3 and 6 month-old GK groups. We included all metabolic parameters measured in these rats to the multivariate regression (Supplementary Fig. 2c, d). Based on this enlarged pattern, we were also able to segregate the cluster of GK rats from Wistar controls (Fig. 8g, h). Using S-plot analysis, we found that blood glucose, visceral fat, and leptin were among the seven most discriminant phenotypic variables for both 3 and 6 month-old GK rats (Fig. 8i, j). Moreover, the most discriminant reproductive parameters were different at 3 months (AFC, LH, AMH, testosterone) and at 6 months (anovulation, testosterone, cysts, LH). In contrast, the metabolic alterations (blood glucose, visceral fat, leptin) kept the same contribution profile at both ages (Fig. 8i, j).

In conclusion, to our knowledge, we describe the only rodent model that spontaneously encapsulates not only the three Rotterdam criteria but also all the other reproductive and metabolic alterations observed in adult and adolescent lean women with PCOS. The study of GK rat gestations and fetuses indicate fetal programming of this PCOS-like phenotype. While we clearly demonstrated deregulation of maternal androgens and of glucose/insulin and AMH levels, further investigations are required to normalize each of these factors in order to determine their respective role in placenta and offspring alterations. In addition, we also showed a strong ovarian response of GK rats to gonadotropin stimulation suggesting that they are also a spontaneous model of OHSS. Finally, a comparison between GK rats and women with PCOS revealed a common reproductive marker signature. The GK rat represents thus an original tool for dissecting the mechanisms involved in the pathogenesis of PCOS, and also for developing novel strategies for treatment and follow-up of this syndrome and its related iatrogenic complications.

## Methods

### Animals

Female GK rats were produced through the mating of male and female type 2 diabetic GK rats in the conventional facility of the Functional and Adaptive Biology Lab (Unité de Biologie Fonctionnelle et Adaptative, Univ de Paris, Paris, France). Non-diabetic Wistar rats were used as controls. All animals were fed ad libitum with a commercial pelleted diet (Diet 113; SAFE; Augy, France). They were group-housed (4 adult rats per cage) under specific pathogen-free conditions in a temperature-controlled room (21–22 °C) with a 12 h–12 h light/darkness cycle and were weaned 28 days after birth.

All procedures were conducted in accordance with the European Community Council directives (2010/63/UE) and approved by the institutional Animal Care and Use Ethical Committee of the Paris-Diderot University (registration number CEEA-40, Agreement B-75-13-17). Adult rats were sacrificed between 3 and 6 months, at the proestrous/estrous stage, following an intraperitoneal injection of pentobarbital (54.7 mg g^−1^of body weight) and 5 postnatal days (pnd) rats and embryonic days (E) 18.5 fetuses by decapitation. All the animals were weighted and measured (tip of the snout−base of the sex), and the Lee Index was calculated (∛body weight per size (g cm^−1^)). The AGD was assessed for 5 pnd rats by measurement of the distance between sex and anus.

### Blood measurements

Blood samples were either collected from the tail for live animals or from the carotid after sacrifice. The plasma was collected in heparin tubes and was centrifuged at 3000 × *g* for 15 min at 4 °C. The serum was extracted from the blood by centrifugation (5 min, 1000 × *g*, 4 °C) after 4 h at 4 °C for coagulation. Because serum volume retrieved on 5 pnd rats was insufficient, samples were pooled (seven pools of two Wistar and five pools of two GK rats). Depending on the assays, total blood, plasma, or serum were used and stored at −80 °C until used.

### Determining glucose and insulin concentrations

Blood glucose concentration was measured using an Accu Check Performa glucometer (Roche, Meylan, France). Plasma insulin levels were determined using a commercial ELISA (Ultrasensitive mouse insulin ELISA, ALPCO Diagnostics, Salem, USA). Glucose tolerance of the rats was assessed by an intraperitoneal glucose injection (1 g kg^−1^ of body weight) performed on 5-month-old Wistar and GK rats in the morning, following 6 h of fasting. Blood glucose and plasma insulin levels were measured prior to the glucose injection, and again after 15, 30, 60, and 90 min.

### Determining leptin, AMH, LH, and estradiol concentrations

Serum leptin levels were measured using the mouse/rat leptin Quantikine ELISA (R&D, Abingdon, UK). Serum AMH levels were determined using the rat and mouse AMH ELISA AL-113 (AnshLabs, Webster, USA). Serum LH levels were measured using the rat LH ELISA (Elabscience, Houston, USA). Serum estradiol concentration was determined using the ultrasensitive estradiol RIA kit (Beckman Coulter, Brea, USA).

### Determining NEFA, HDL- and LDL-C and TG concentrations

Non-esterified fatty acids (NEFA) were measured in rat serum using the NEFA-HR (2) kit (Wako diagnostics, Moutain View, USA). HDL- and LDL-cholesterol (C) and TG levels were measured in rat plasma using, the HDL-C and LDL-C L-Type kits (Wako diagnostics) and the Triglycerides-LQ kit (SOBIODA, Montbonnot-Saint-Martin, France), respectively.

### Quantification of steroids

Progesterone, testosterone, and delta-4 androstenedione were measured in rat serum and in women follicular fluid (FF) by serum liquid chromatography coupled to tandem mass spectrometry (LC–MS/MS)^37^. Briefly, a mixture of the deuterated internal standard (150 µL) was added to 50–100 µL of serum or FF. The samples were allowed to adsorb for 5 min on support liquid extraction columns (Isolute SLE+, Biotage, Uppsala, Sweden) before elution of the steroids through the addition of 0.9 mL (twice) methylene chloride. The eluates, which contained the non-conjugated steroids, were evaporated until dry and reconstituted to 150 µL in methanol and water (50:50).

Steroids were chromatographically separated by high-performance liquid chromatography using a Shimadzu Nexera XR system (Shimazu France, Marne la Vallee, France) and a Coreshell C18 column (Kinetex, 2.6 µm 100 Å, 100 per 2.1 mm; Phenomenex, Le Pecq, France). Detection was performed using a triple quadripole mass spectrometer (Triple Quad 6500, ABSciex, Foster City, CA, USA). Upon collection, the LC–MS/MS data were analyzed using MultiQuant software (ABSciex, Foster City, CA, USA version 3.0) with built-in queries or quality control rules allowing us to set compound-specific criteria for flagging outlier results. Flagging criteria included accuracies for standards and quality controls, quantifier ion per qualifier ion ratios, and lower and upper calculated concentration limits. For each calibration curve, the regression line used for quantitation was calculated using least-squares weighting.

### Body composition and fat mass analysis

Body composition was analyzed on 3-month-old Wistar and GK rats by EcoMRI™ 100 (Whole Body Analyzers, EchoMRI, Houston, USA). Lean mass and fat mass were determined for each rat. Total inguinal, perirenal, periovarian, and parametrial fat pads were cautiously dissected and weighed. All results are expressed as the percentage of total body weight.

### Assessment of the estrous cycle

From 4 to 8 weeks of age, rats were monitored to determine the day of their vaginal opening. Briefly, a glass swab was moistened with physiological serum and inserted in the rat’s vagina. The cells were quickly spread on a slide and the smear was left at room temperature until totally dry. The cells were stained with Giemsa (Sigma-Aldrich, Saint Louis, USA) (dilution 1:10), washed with tap water and examined under optical microscope to determine estrous cycle changes^69^.

### Ovarian histology

#### Corpora lutea, cysts, and atretic follicles measurements

Ovaries were fixed 24 h at 4 °C in BOUIN solution (10% paraformaldehyde (PFA); 15% picric acid; 5% acetic acid). Paraffin-embedded ovaries were sectioned at a thickness of 5 μm and stained with hematoxylin–eosin (Sigma Aldrich, Saint-Quentin Fallavier, France).

Cysts were defined as dilated follicles containing cavities filled with follicular fluid, and lined with one to five cell layers thick of round-to-flattened GC^59^. The corpora lutea and cysts count was performed under optical microscope using a × 20 objective on 1 ovary section per 10. To avoid redundant counting, every cyst-like follicle was counted only in the section where the oocyte’s nucleus was visible and the number of corpora lutea was determined through a comparison with the preceding and following sections. The surface of every ovary section was measured using the Histolab software version 10.5.0.1 (Microvision Instruments, Evry, France), and results are expressed by corpora lutea or cysts per mm^3^ of ovary. Theca and GC layers were defined by examination of cell morphology. The area of theca cells was measured using the Histolab software, and results are expressed as the percentage of theca cells layer area of the antral follicle.

With regard to atretic follicles measurements, antral follicles were considered atretic if they displayed pycnotic GC within the antral cavity and a degenerating oocyte with no connection with the cumulus GC^70^. Results are expressed as the percentage of atretic follicles found among the antral follicles.

#### Follicles count

Ovaries were fixed for 24 h at 4 °C in 4% PFA and paraffin-embedded ovaries were sectioned at a thickness of 5 μm. After antigen retrieval (10 min, three times at 750 W in 10 mM citrate buffer, pH 6), sections were incubated for 15 min with 0.3% H_2_O_2_ in pure methanol. Non-specific sites were blocked with 5% horse serum in 1 × Dulbecco’s phosphate-buffered saline (DPBS) (Gibco, ThermoFisher Scientifc, Waltham, USA) for 30 min, and the sections were incubated overnight at 4 °C with a monoclonal mouse anti-proliferating cell nuclear antigen (PCNA) antibody (Dako, Santa Clara, USA, M0879; 1:250 dilution). After two DPBS washes, sections were incubated with an anti-mouse biotinylated secondary antibody (Vectastain Universal Anti Mouse IgG/Rabbit IgG ABC kit, Vector Laboratories, Peterborough, United Kingdom, BA-1300; 1:200 dilution) at room temperature for 1 h. The slides were incubated for 30 min with the streptavidin–biotin–peroxidase complex after two DPBS washes. Peroxidase activity was developed using 3,3-diaminobenzidine tetrahydrachloride (Peroxidase Substrate kit, Vector Laboratories). Finally, all sections were counterstained with hematoxylin.

The follicles count was performed under optical microscope using a × 20 objective on 1 ovary section per 10. They were allocated to four classes: primordial follicles containing a partial or flattened GC encircling the oocyte; primary follicles with an oocyte surrounded by a single layer of cuboidal GC; preantral follicles comprising 2–6 GC layers; and antral follicles containing an antral cavity. Only the follicles with a visible oocyte were counted. Results are expressed as follicles per mm^3^ of ovary as described above.

#### Adipocyte size measurement

Samples of periovarian adipose tissue collected on 3-month-old Wistar and GK rats were fixed for 24 h at 4 °C in BOUIN solution. Paraffin-embedded samples were sectioned at 5 μm thickness and stained with hematoxylin–eosin. Adipocyte size distribution was determined on 5–10 fields of periovarian adipose tissue per section covering the entire tissue surface, at original magnification 310 (optical microscope IX83, Olympus). Adipocyte surface quantification was performed using ImageJ software (win 6 4) (hppt://rsbweb.nih.gov/ij/) on 1000–5000 cells per rat.

#### RNA extraction and RT-qPCR analysis

Tissues were collected, snap-frozen in liquid nitrogen, and stored at −80 °C. For adipose tissue, total RNA was isolated by Qiazol extraction (Qiagen, Courtaboeuf, France) and purified using Qiagen RNeasy Plus mini Kit (Qiagen). For the ovaries and placenta, total RNA was directly purified using Qiagen RNeasy Plus mini Kit. 500 ng of RNA were retrotranscribed using the High Capacity cDNA RT kit (Applied Biosystem, Foster City, USA). Real-time PCR was performed using the Taqman method, in duplicates with one-fifth dilution of the cDNA using the LightCycler 480 Probes Master kit (Roche Diagnostics, Meylan, France). The primers and the Universal ProbeLibrary probes (Roche Diagnostics, Indianapolis, USA) used to amplify these genes are indicated in the Supplementary Table 2. The PCR protocol used an initial denaturation step at 95 °C for 10 min followed by 45 cycles of 95 °C for 10 s, 60 °C for 30 s, 72 °C for 1 s. PCR products of each gene of interest were purified and quantified to generate external standard curves ranging from 10^2^ to 10^7^ copies μL^−1^. Data were obtained as copy numbers, normalized according to the expression of the housekeeping gene hypoxantine-guanine phospho-ribosyl-transferase (HPRT). Values are expressed relative to control values (Wistar rats), set at 1.

#### Ovarian gonadotropin stimulation

GK and Wistar rats were first injected intraperitoneally with 10 IU of PMSG (Sigma, St. Louis, MO) and after 48 h with 10 IU of hCG. Sixteen hours later, animals were killed by decapitation and hematocrit measures were determined directly by microhematocrit centrifugation. The cumulus-oocytes were extracted from the fallopian tubes and the oocytes were counted after hyaluronidase (80 IU ml^−1^; 10 min) treatment for decoronization.

#### Subjects

Women undergoing in vitro fertilization were included in this study according to their clinical parameters captured by clinicians in Medifirst-AMP software (version 1.4.8.5, Montigny-le-Bretonneux, France), 44 in the control group and 45 in the PCOS group. All women in the control group had BMI < 25 kg m^−2^ and met the following inclusion criteria: (1) age between 20 and 40; (2) both ovaries present, with no morphological abnormalities, adequately visualized by transvaginal ultrasound scans; (3) menstrual cycle length range between 26 and 30 days; (4) no current or past diseases affecting the ovaries or gonadotropin and sex steroid secretion, clearance, or excretion; (5) no clinical signs of hyperandrogenism; and (6) no polycystic ovary morphology at ultrasonography. Infertility was due either to tubal or sperm abnormalities. Women with PCOS included in this study presented the two out of the three Rotterdam criteria namely, ovulatory disturbances, polycystic ovary morphology, and hyperandrogenism and a BMI < 25 kg m^−2^. This investigation received the approval of the Tenon Hospital Internal Institutional Review Board (CPP PREFENDO 18.10.58, PI Emmanuelle Mathieu d’Argent) and all women signed an informed consent before participating. The study was conducted in accordance to the criteria set by the Declaration of Helsinki.

#### Statistics and reproducibility

No statistical methods were used to predetermine the experimental sample size. Sample size (number of biologically independent rats or women, *n*), *p*-values and the types of statistical tests are all indicated on the figures or in figure legends. Only female mice were used for experiments and were allocated so that each group was evenly matched for age range as indicated on the figures or in figure legends. Data were first subjected to normality test and then to two-sided Mann–Whitney *U* tests followed by a post-hoc Bonferroni’s adjustment for Multiple group comparisons (Wistar and GK rats) using the GraphPad Prism software version 6.01 for Windows (GraphPad Software, La Jolla, CA, USA). Differences were considered significant at *p* < 0.05. The *p*-values are provided in the figures and in Source Data files; only the exact *p*-values > 0.0001 were calculated by the GraphPad Prism software. *χ*^2^ analysis was performed to study the statistical differences in adipocyte surface distribution.

Multivariate data analysis was performed using SIMCA software (version 15, MKS Umetrics AB, Sweden). Logarithmic transformation was used systematically to minimize the impact of both noise and high variance of some variables. Data were then implemented for unsupervised principal components analysis (PCA) to identify similarities or differences between sample profiles. Secondarily, supervised statistical methods using projections to latent structures (PLS) and orthogonal projections to latent structures (OPLS) regression were achieved. PLS modeling techniques are suitable for our data matrix since they can handle multiple variable systems with small sample size. Simultaneously, these approaches are also able to support multicollinearity and missing values. Orthogonal partial least-squares discriminant analysis (OPLS-DA) uses a binary variable for *Y* that represents class membership (in our case women with PCOS vs. control women or GK vs. Wistar rats). Predictions have a value between 0 and 1 depending on class membership. The OPLS-DA model indicates which are the driving forces among the variables. Score plots help to visualize the differences between the observations (if they exist) and generally the horizontal component of the OPLS-DA score scatter plot captures variations between the groups and the vertical dimension captures variations within the groups. Loading plots indicate the variables that express these particular differences. OPLS-DA tells which variables have the largest discriminatory power and how well the variables are correlated. It will also quantify how much of the variation in the *X* block (metabolic and/or reproductive criteria) is actually relevant to the analyzed question.

### Reporting summary

Further information on research design is available in the Nature Research Reporting Summary linked to this article.

## Supplementary information

Supplementary Information

Reporting Summary

## Data Availability

The authors declare that all the data supporting the findings of this study are available within the paper. Source data are provided with this paper.

## Source data

Source Data
