# Supplementary material for: The Goto-Kakizaki rat is a spontaneous prototypical rodent model of polycystic ovary syndrome
Source: Nat Commun. 2021 Feb 16;12:1064. doi: 10.1038/s41467-021-21308-y (PMC7886868; doi:10.1038/s41467-021-21308-y)
Supplement: Supplementary file 1 — Supplementary Information [file 41467_2021_21308_MOESM1_ESM.pdf]

The Goto-Kakizaki rat is a spontaneous prototypical rodent model of the polycystic ovary syndrome

Camille Bourgneuf[1,2], Danielle Bailbe[3], Antonin Lamazière[1,4], Charlotte Dupont[1,2,5], Marthe Moldes[1,2], Dominique Farabos[1,4], Natacha Roblot[1,2], Camille Gauthier[1,2], Emmanuelle Mathieu d'Argent[1,2,5], Joelle Cohen-Tannoudji[3], Danielle Monniaux, Bruno Fève[1,2,7], Jamileh Movassat[3], Nathalie di Clemente[1,2], Chrystèle Racine[1,2,8,*]

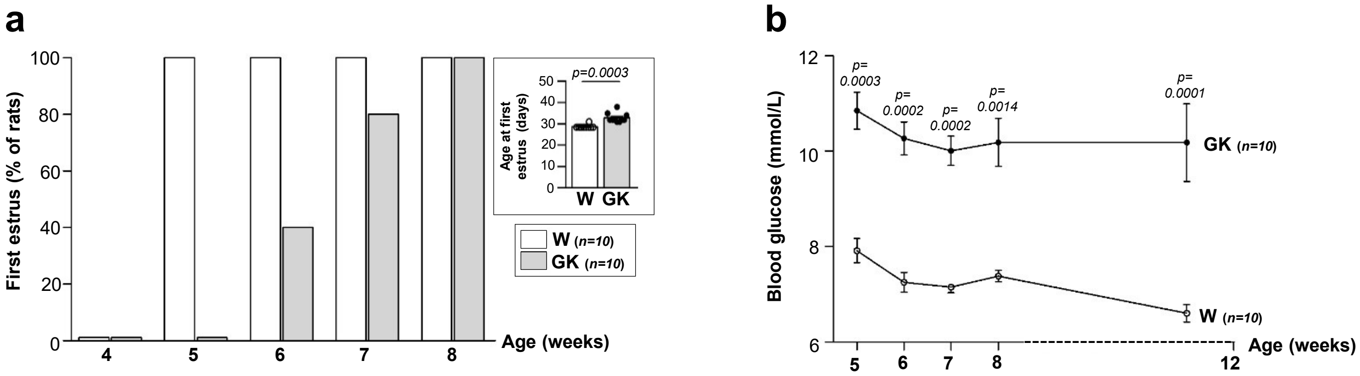

Supplementary Figure 1. Study of pubertal development of Wistar and GK rats. **a**. Percentage of rats (Wistar: white bars, GK: grey bars) with first estrus on various postnatal weeks up to week 8. Inset shows the mean age of the rats at first estrus. **b** Evolution of blood glucose measured on 5 to 8 weeks-old Wistar (open symbols) and GK (closed symbols) rats. Data are represented as mean ± SEM. *P*-values are from two-sided Mann Whitney U test for unpaired comparisons (GK vs. Wistar rats) followed by Bonferroni's adjustment. *n* represents the number of biologically independent animals in each group. Source data are provided as a Source Data file.

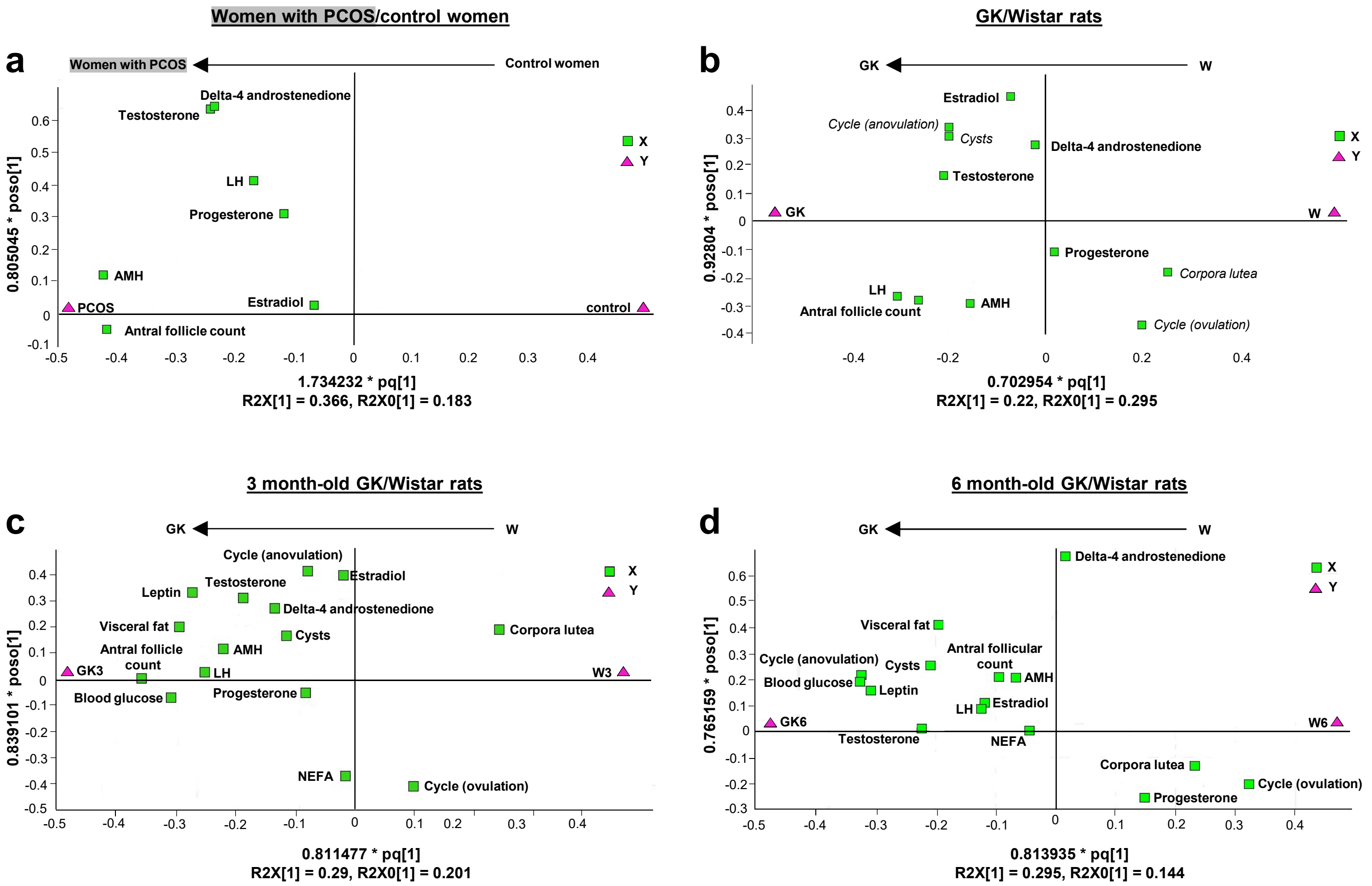

Supplementary Figure 2. Orthogonal partial least-squares discriminant analysis. **a** Orthogonal partial least-squares discriminant analysis (OPLS-DA) load plot based on 7 reproductive criteria (Antral follicle count, serum AMH, LH and estradiol levels (**Supplementary Table 1**), intrafollicular fluid levels of testosterone, delta-4 androstenedione, progesterone) in women with PCOS and control women. **b** OPLS-DA load plot based on 11 reproductive criteria (Antral follicle count, serum AMH, LH, estradiol, testosterone, delta-4androstenedione, progesterone levels, corpora lutea, ovulation/anovulation) in GK and Wistar rats. **c,d** OPLS-DA load plot based on 15 metabolic and reproductive criteria (Antral follicle count, serum AMH, LH, estradiol, testosterone, delta-4-androstenedione, progesterone levels, corpora lutea, ovulation/anovulation, blood glucose, NEFA, visceral fat, leptin) in GK and Wistar rats at 3 (**c**) and 6 (**d**) months.

		control women (n=44) Med (25-75th)	Women with PCOS (n=45) Med (25-75th)	*p*
Clinical parameters	Age (years)	34 (32-37)	32 (28-35)	*P*=0.0055
	BMI (kg/m²)	21.9 (20.1-23.4)	21.4 (19.9-22.8)	ns
	Day 3 AFC	15 (12-18)	45 (33-54)	*p*<0.0001
Serum (day 3)	E2 (pg/mL)	41.5 (28.8-51.6)	47.2 (31-55)	ns
	LH (UI/L)	5.3 (4-6.6)*	8.0 (5.1-9.6)*	*p*=0.0003
	FSH (UI/L)	7.6 (6.4-8.9)	5.5 (4.6-6.9)	*p*<0.0001
	LH/FSH ratio	0.7 (0.5-0.9)	1.6 (1-1.6)	*p*<0.0001
	AMH (ng/mL)	2.4 (1.7-3.1)	11.6 (7.1-12.7)	*p*<0.0001

Supplementary Table 1. Main clinical parameters in control women and women with PCOS. Results were analyzed by Mann-Whitney U test for unpaired comparisons between control women and women with PCOS. Abbreviations: BMI, body mass index; AFC, total antral follicle count; E2, 17β-estradiol; LH, luteinizing hormone; FSH, follicle stimulating hormone; AMH, anti-Müllerian hormone; day 3, hormonal dosage on day 3 of the menstrual cycle; Med, median (25-75th percentile); ns, non significant. n=42 for control women and n=43 for women with PCOS.

	Nucleotide sequence 5' --> 3'		Library Probe (Universal Probe)	NCI reference sequence	Length (bp)	Efficiency (%)
Gene	Forward	Reverse				
Amh	GGAGAGACTGGGGAACAGC	CAAGAGCTGAGGCTCCCATA	41	NM_012902	69	98
Amhr2	CAACATCCCTTCCTCTTGGAG	CGTCCCAGCAATCTTCCA	53	NM_030998	77	96
Hprt	GGTCCATTCCTATGACTGTAGATTTT	CAATCAAGACGTTCTTTCCAGTT	22	NM_012583	126	95
Gpat	TCCAGACACCACATCAAGGA	ATTGGGCAGATAAGAAACGTCTA	25	NM_017274.1	112	98
Dgat	CCGTGGTATCCTGAATTGGT	AAAGATAACCTTGCATTACTCAGGA	9	NM_053437.1	60	96
Acaca	GCTCAGATACACTTTCTGATTTGG	TAGGTGCAAGCCGGACAT	13	NM_022193.1	89	97
Fasn	TCGTCTGCCTCCAGATCC	GGCAATTTCCCGGACATAC	25	NM_017332.1	75	96
Pparγ	CAGGAAAGACAACAGACAAATCA	GGGGGTGATATGTTTGAACTTG	7	NM_013124.3	95	97
Lipe	CCGAGCACTGGAGGAGTG	ATATCCGCTCTCCGGTTGA	107	NM_012859.1	83	96
Leptin	CCAGGATCAATGACATTTCACA	AATGAAGTCCAAACCGGTGA	13	NM_013076.3	71	121
Hsd3b1	GAGGATCGTCCAGTTGTTGG	TTGATGCTTGTCCCTAGGTTG	2	L17138.1	114	114

Supplementary Table 2. Sequence of the primers and probes used for real time RT-PCR experiments
