# Supplementary material for: The Goto-Kakizaki rat is a spontaneous prototypical rodent model of polycystic ovary syndrome
Source: Nat Commun. 2021 Feb 16;12:1064. doi: 10.1038/s41467-021-21308-y (PMC7886868; doi:10.1038/s41467-021-21308-y)
Supplement: Supplementary file 2 — Reporting Summary [file 41467_2021_21308_MOESM2_ESM.pdf]

nature research

Corresponding author(s): Chrystele Racine

Last updated by author(s): Dec 16, 2020

Reporting Summary

Nature Research wishes to improve the reproducibility of the work that we publish. This form provides structure for consistency and transparency in reporting. For further information on Nature Research policies, see Authors & Referees and the Editorial Policy Checklist .

Statistics

For all statistical analyses, confirm that the following items are present in the figure legend, table legend, main text, or Methods section.

n/a	Confirmed	
☐	☒	The exact sample size (*n*) for each experimental group/condition, given as a discrete number and unit of measurement
☐	☒	A statement on whether measurements were taken from distinct samples or whether the same sample was measured repeatedly
☐	☒	The statistical test(s) used AND whether they are one- or two-sided<br>*Only common tests should be described solely by name; describe more complex techniques in the Methods section.*
☐	☒	A description of all covariates tested
☐	☒	A description of any assumptions or corrections, such as tests of normality and adjustment for multiple comparisons
☐	☒	A full description of the statistical parameters including central tendency (e.g. means) or other basic estimates (e.g. regression coefficient) AND variation (e.g. standard deviation) or associated estimates of uncertainty (e.g. confidence intervals)
☐	☒	For null hypothesis testing, the test statistic (e.g. *F*, *t*, *r*) with confidence intervals, effect sizes, degrees of freedom and *P* value noted<br>*Give P values as exact values whenever suitable.*
☒	☐	For Bayesian analysis, information on the choice of priors and Markov chain Monte Carlo settings
☒	☐	For hierarchical and complex designs, identification of the appropriate level for tests and full reporting of outcomes
☐	☒	Estimates of effect sizes (e.g. Cohen's *d*, Pearson's *r*), indicating how they were calculated

Our web collection on statistics for biologists contains articles on many of the points above.

Software and code

Policy information about availability of computer code

Data collection	- Medifirst-AMP version 1.4.8.5 (Montigny-le-Bretonneux, France)
Data analysis	- GraphPad Prism version 5.00 for Windows (GraphPad Software, La Jolla, California, USA)<br>- SIMCA software version 15 (MKS Umetrics AB, Sweden)<br>- Histolab software version 10.5.0.1 (Microvision Instruments, Evry, France)<br>- ImageJ software (win 6 4)(hppt://rsbweb.nih.gov/ij/)<br>- MultiQuant software version 3.0 (ABSciex, Foster City, CA, USA)

For manuscripts utilizing custom algorithms or software that are central to the research but not yet described in published literature, software must be made available to editors/reviewers. We strongly encourage code deposition in a community repository (e.g. GitHub). See the Nature Research guidelines for submitting code & software for further information.

Data

Policy information about availability of data

All manuscripts must include a data availability statement. This statement should provide the following information, where applicable:

- Accession codes, unique identifiers, or web links for publicly available datasets
- A list of figures that have associated raw data
- A description of any restrictions on data availability

All the data supporting the findings of this study are available within the paper and its supplementary information files. Raw data associated to these finfings are provided in Source data files.

Field-specific reporting

Please select the one below that is the best fit for your research. If you are not sure, read the appropriate sections before making your selection.

[x] Life sciences   [ ] Behavioural & social sciences   [ ] Ecological, evolutionary & environmental sciences

For a reference copy of the document with all sections, see nature.com/documents/nr-reporting-summary-flat.pdf

Life sciences study design

All studies must disclose on these points even when the disclosure is negative.

Sample size	The sample size was not predetermined. We used the current standard for rat experiments, based on the minimal amount of rat required to detect significance with an alpha rate set at 0.05 in a standardly powered experiment, taking into consideration the reproductive capacity of GK rats. We used all the women that had been included in the CPP Prefendo at the time of the PLS analysis and that fullfilled the inclusion criteria of our study (n=44 for control women and n=45 for women with PCOS). Results of the PLS analysis showed retrospectively that the sample size was sufficient to segregate control women from women with PCOS.
Data exclusions	Only 1 out of 9 insulin concentrations was excluded for Wistar rats at 3 months (Fig. 1b) because it was undetectable.
Replication	Each experiment presented in the paper was repeated in multiple biologically independent animals (between 5 and 24 per experiment) and women (n>44).
Randomization	No randomization was needed for grouping of rats or women into experimental groups. Female rats were age and phenotype (Goto-Kakisaki and Wistar) matched. Women (control or with PCOS) were grouped according to their clinical parameters.
Blinding	Rat blinding was not relevant to the study, as rats were assigned to group based on their phenotype (Wistar or GK), and for histological analyses measurements of ovarian criteria were performed unknowing the phenotype of the rat. Patient blinding was not relevant to the study, as women were assigned to groups by clinicians based on their clinical parameters.

Reporting for specific materials, systems and methods

We require information from authors about some types of materials, experimental systems and methods used in many studies. Here, indicate whether each material, system or method listed is relevant to your study. If you are not sure if a list item applies to your research, read the appropriate section before selecting a response.

Materials & experimental systems

n/a	Involved in the study	
[ ]	[x]	Antibodies
[x]	[ ]	Eukaryotic cell lines
[x]	[ ]	Palaeontology
[ ]	[x]	Animals and other organisms
[ ]	[x]	Human research participants
[x]	[ ]	Clinical data

Methods

n/a	Involved in the study	
[x]	[ ]	ChIP-seq
[x]	[ ]	Flow cytometry
[x]	[ ]	MRI-based neuroimaging

Antibodies

Antibodies used	This work used monoclonal mouse anti-PCNA antibody (Dako, Santa Clara, USA; Cat.M0879; Clone # PC10) at a 1:250 dilution and anti-mouse biotinylated secondary antibody (Vectastain Universal Anti Mouse IgG/Rabbit IgG ABC kit, Vector Laboratories, Peterborough, United Kingdom, BA-1300) at 1:200 dilution.
Validation	Although ovarian follicle counting has been traditionally performed manually on hematoxylin and eosin-stained sections, the use of immunohistochemical methods proliferating cell nuclear antigen (PCNA) immunohistochemistry has been used to enhance the visibility of the primordial and primary follicles to facilitate manual counting (Picut et al, 2008, toxicologic pathology, 36).

Animals and other organisms

Policy information about studies involving animals; ARRIVE guidelines recommended for reporting animal research

Laboratory animals	Female Goto-Kakizaki rats used in this study were issued from the Paris colony (GK/Par line). Female nondiabetic Wistar rats were used as controls. The rats were sacrificed at 3 and 6 months.
Wild animals	The study did not involve wild animals.

Field-collected samples	The study did not involve samples collected from the field.
Ethics oversight	All procedures were conducted in accordance with the European Community Council directives (2010/63/UE) and approved by the institutional Animal Care and Use Ethical Committee of the Paris-Diderot University (registration number CEEA-40, Agreement B-75-13-17).

Note that full information on the approval of the study protocol must also be provided in the manuscript.

Human research participants

Policy information about studies involving human research participants

Population characteristics	Women undergoing in vitro fertilization were included in this study, 44 in the control group and 45 in the PCOS group. All the women were lean (BMI < 25 kg/m2) and between 28-37 years old.
Recruitment	There are no biaises of selection of the women because we used all the women that had been included in the CPP Prefendo at the time of the PLS analysis and that fullfilled the inclusion criteria of our study. These inclusion criteria are based on the clinical parameters of the women captured by the clinicians. The women allocated to the control group by the clinicians met the following inclusion criteria: 1) age between 20 and 40; 2) both ovaries present, with no morphological abnormalities, adequately visualized by transvaginal ultrasound scans; 3) menstrual cycle length range between 26 and 30 days; 4) no current or past diseases affecting the ovaries or gonadotropin and sex steroid secretion, clearance, or excretion; 5) no clinical signs of hyperandrogenism; and 6) no polycystic ovary morphology at ultrasonography. Infertility was due either to tubal or sperm abnormalities. Women with PCOS included in this study presented the three Rotterdam criteria namely, ovulatory disturbances, polycystic ovary morphology and hyperandrogenism. All women had a BMI<25 kg/m2.
Ethics oversight	The investigation received the approval of the Committee of person protection Ile de France III (CPP PREFENDO 18.10.58, PI Emmanuelle Mathieu d'Argent) and all women signed an informed consent before participating.

Note that full information on the approval of the study protocol must also be provided in the manuscript.
